# Glucose fluxes in glycolytic and oxidative pathways detected *in vivo* by deuterium magnetic resonance spectroscopy reflect proliferation in mouse glioblastoma

**DOI:** 10.1016/j.nicl.2021.102932

**Published:** 2022-01-05

**Authors:** Rui V. Simões, Rafael N. Henriques, Beatriz M. Cardoso, Francisca F. Fernandes, Tânia Carvalho, Noam Shemesh

**Affiliations:** Champalimaud Research, Champalimaud Centre for the Unknown, Lisbon, Portugal

**Keywords:** Deuterium magnetic resonance spectroscopy, Glioblastoma, Glycolysis, Mitochondrial oxidation, Magnetic resonance imaging

## Abstract

**Objectives:**

Glioblastoma multiforme (GBM), the most aggressive glial brain tumors, can metabolize glucose through glycolysis and mitochondrial oxidation pathways. While specific dependencies on those pathways are increasingly associated with treatment response, detecting such GBM subtypes *in vivo* remains elusive. Here, we develop a dynamic glucose-enhanced deuterium spectroscopy (DGE ^2^H-MRS) approach for differentially assessing glucose turnover rates through glycolysis and mitochondrial oxidation in mouse GBM and explore their association with histologic features of the tumor and its microenvironment.

**Materials and methods:**

GL261 and CT2A glioma allografts were induced in immunocompetent mice and scanned *in vivo* at 9.4 Tesla, harnessing DGE ^2^H-MRS with volume selection and Marchenko-Pastur PCA (MP-PCA) denoising to achieve high temporal resolution. Each tumor was also classified by histopathologic analysis and assessed for cell proliferation (Ki67 immunostaining), while the respective cell lines underwent *in situ* extracellular flux analysis to assess mitochondrial function.

**Results:**

MP-PCA denoising of *in vivo* DGE ^2^H-MRS data significantly improved the time-course detection (~2-fold increased Signal-to-Noise Ratio) and fitting precision (−19 ± 1 % Cramér-Rao Lower Bounds) of ^2^H-labelled glucose, and glucose-derived glutamate-glutamine (Glx) and lactate pools in GL261 and CT2A orthotopic tumors. Kinetic modeling further indicated inter-tumor heterogeneity of glucose consumption rate for glycolysis and oxidation during a defined epoch of active proliferation in both cohorts (19 ± 1 days post-induction), with consistent volumes (38.3 ± 3.4 mm^3^) and perfusion properties prior to marked necrosis. Histopathologic analysis of these tumors revealed clear differences in tumor heterogeneity between the two GBM models, aligned with metabolic differences of the respective cell lines monitored *in situ*. Importantly, glucose oxidation (i.e. Glx synthesis and elimination rates: 0.40 ± 0.08 and 0.12 ± 0.03 mM min^−1^, respectively) strongly correlated with cell proliferation across the pooled cohorts (R = 0.82, p = 0.001; and R = 0.80, p = 0.002, respectively), regardless of tumor morphologic features or *in situ* metabolic characteristics of each GBM model.

**Conclusions:**

Our fast DGE ^2^H-MRS enables the quantification of glucose consumption rates through glycolysis and mitochondrial oxidation in mouse GBM, which is relevant for assessing their modulation *in vivo* according to tumor microenvironment features such as cell proliferation. This novel application augurs well for non-invasive metabolic characterization of glioma or other cancers with mitochondrial oxidation dependencies.

## Highlights

• We performed dynamic glucose enhanced (DGE) ^2^H-MRS in mouse GBM tumors.
• Marchenko-Pastur PCA denoising of ^2^H-MRS spectra improved kinetic quantification.
• Metabolic kinetics revealed differential glucose pathway fluxes in non-necrotic tumors.
• Modulation of glucose metabolism reflected tumor heterogeneity (proliferation).

## 1 Introduction

Cancer is a highly heterogeneous disease, exhibiting multiple phenotypes that impose major challenges for clinical diagnosis and treatment, including assessment of treatment efficacy. Tumor heterogeneity can be characterized by many different aspects, such as cell proliferation, invasion, and hypoxia. However, recent observations suggest that a crucial feature of tumor heterogeneity involves the underlying metabolism (Cantor and Sabatini, 2012). While aerobic glycolysis is a well-established hallmark of cancer metabolism (Warburg effect (Warburg, 1956)), oxidation of glucose through the mitochondrial tricarboxylic acid cycle (TCA) pathway is becoming increasingly associated with microenvironment adaptation and tumor progression (Faubert et al., 2020). Such *metabolic heterogeneity* is observed in glioblastoma multiforme (GBM), the most aggressive form of glial brain tumors (grade IV) (Wen and Kesari, 2008). Specifically, the tumor’s proclivity to metabolize glucose through glycolysis and mitochondrial oxidation (Maher et al., 2012) is now being associated with pathway-specific dependencies for different GBM subtypes and their respective vulnerabilities to targeted treatments. Namely, a so-called mitochondrial GBM subtype, with cellular bioenergetics relying exclusively on oxidative phosphorylation (OXPHOS), has demonstrated the highest sensitivity to OXPHOS inhibition and most favorable clinical outcome (Garofano et al., 2021). Moreover, different metabolic ”rewiring” of glycolysis and oxidative metabolic pathways were recently reported in subpopulations of human GBM cells (U87MG) as reflected by their temozolomide resistance, the current gold-standard for adjuvant chemotherapy in GBM (Immanuel et al., 2021). Accordingly, targeting mitochondrial metabolism for cancer therapy (Weinberg and Chandel, 2015) is currently being pursued with new treatment modalities in GBM (Molina et al., 2018, Shi et al., 2019).

Measuring metabolic fluxes in glycolysis and mitochondrial oxidation pathways simultaneously could thus harbinger metabolic phenotyping and early assessment of treatment efficacy. However, such characterization remains elusive due to a paucity of (invasive and non-invasive) methods with sufficient sensitivity and specificity towards properties associated with such metabolic fluxes. For example, non-invasive molecular methods such as ^18^FDG-PET can only detect tumor glucose uptake, but not its metabolic turnover. *In vitro* analysis of biopsy samples collected after *in vivo* administration of glucose tracers (e.g., ^13^C-labelled) represents an invasive approach for detecting glycolytic and oxidative metabolism of glucose but not their respective *in vivo* fluxes, in clinical and preclinical GBM tumors (Maher et al., 2012, Marin-Valencia et al., 2012). ^1^H magnetic resonance spectroscopy (^1^H-MRS) provides noninvasive information for tumor metabolic profiling, making it possible to distinguish relevant GBM subtypes such as IHD-mut (Choi et al., 2012). However, it is unlikely that it could be used directly to assess metabolic turnover rates due to the crowded spectral areas. ^13^C magnetic resonance spectroscopy (^13^C-MRS) can overcome this limitation to some extent by detecting the non-toxic carbon isotope in labeled glucose. Indeed, ^13^C-MRS with infused ^13^C-glucose tracers has been proposed for measuring glucose fluxes through glycolysis and mitochondrial oxidation *in vivo* in gliomas; however, these advanced methods have mostly been limited to very large voxel sizes, with significant non-tumor tissue contributions, as shown in human GBM (Wijnen et al., 2010) and patient-derived GBM xenografts (Lai et al., 2018). Furthermore, the temporal dynamic range of ^13^C-MRS is inherently very low due to the long carbon longitudinal relaxation constants (Lai et al., 2018).

Deuterium magnetic resonance spectroscopy (^2^H-MRS) is a highly promising recently developed MRS modality based on intra-venous injection of non-toxic ^2^H-labelled substrates, including ^2^H-glucose. Compared to its ^1^H-MRS counterpart, ^2^H-MRS benefits from: (i) no detectable metabolic background signals, facilitating specificity and sensitivity (the natural abundance of deuterium is very low, and typically only trace amounts are observed from the ∼ 110 M proton signal in pure water); (ii) short metabolite longitudinal relaxation times, rapid sampling of the signals; and (iii) an internal reference for quantification that does not require pre-saturation – naturally abundant semi-heavy water, DHO (Lu et al., 2017). Recent studies have demonstrated the potential of ^2^H-MRS coupled with ^2^H-glucose injection for tumor metabolic imaging, proving a non-invasive quantitative assessment of the Warburg effect in clinical and preclinical GBM (De Feyter et al., 2018) and early therapeutic response monitoring in mouse subcutaneous tumors (Kreis et al., 2020), also reported with ^2^H-fumarate injection (Hesse et al., 2021). Importantly, Dynamic Glucose-Enhanced (DGE) ^2^H-MRS has been used to measure glucose consumption rates in the normal rat brain, linked to mitochondrial oxidation (Lu et al., 2017), and glycolysis rates in a mouse lymphoma model (Kreis et al., 2020). Therefore, we hypothesized the suitability of DGE ^2^H-MRS for detecting both pathway fluxes simultaneously in GBM tumors.

Here, we selected two well-established syngeneic models of GBM – GL261 and CT2A – recapitulating histologic, genetic, and immunogenic features of the disease (Martinez-Murillo and Martinez, 2007, Oh et al., 2014, Seligman and Shear, 1939, Seyfried et al., 1992, Zagzag et al., 2000), and harnessed a novel application of DGE ^2^H-MRS with volume selection and spectral denoising based on Marchenko-Pastur PCA (MP-PCA) (Veraart et al., 2016) to: (i) demonstrate its ability to measure glucose consumption rates through glycolysis and mitochondrial oxidation in mouse GBM *in vivo*; and (ii) explore potential modulations of glucose metabolism according to GBM microenvironment features, such as cell proliferation (Anderson and Simon, 2020, Charles et al., 2012).

## 2 Materials and methods

**Fig. 1:**
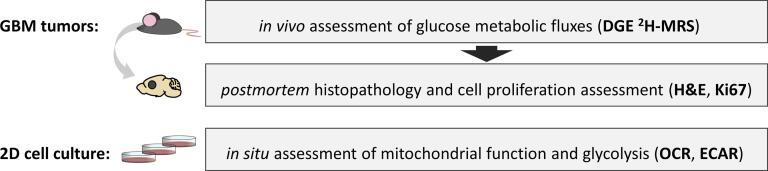
**Experimental design**. Two mouse allograft models were used to dynamically assess glucose metabolism in GBM tumors *in vivo*, using DGE ^2^H-MRS under regular anesthesia conditions: GL261 (n = 7) and CT2A (n = 5). GBM-bearing mice were sacrificed, and the fixed tumors further analyzed *post-mortem* (n = 9) by histopathologic classification (H&E staining) and quantification of cell proliferation (Ki67 immunostaining). Additionally, GL261 and CT2A cancer cell metabolism were assessed *in situ*, during active cell proliferation in 2D culture, based on extracellular flux analysis under basal conditions followed by respiration inhibition (n = 3).

As summarized in Fig. 1, this study included *in vivo* and *post-mortem* assessment of mouse GBM tumors, namely the GL261 and CT2A models, which were complemented by *in situ* analysis of these cell lines.

### 2.1 Animals and cell line

All animal experiments were preapproved by institutional and national authorities and carried out according to European Directive 2010/63. A total of n = 13 C57BL/6j mice were used in this study, bred at the Vivarium of the Champalimaud Foundation, and housed with *ad libitum* access to food and water and normal light cycles. GL261 mouse glioma cells were obtained from the Tumor Bank Repository at the National Cancer Institute (Frederick MD, USA). CT2A mouse glioma cells were kindly provided by Prof. Thomas Seyfried at Boston College (Boston MA, USA)). Both cell lines were grown in RPMI-1640 culture medium supplemented with 2.0 g/l Sodium Bicarbonate, 0.285 g/l L-glutamine, 10 % Fetal Bovine Serum (Gibco) and 1 % Penicillin-Streptomycin solution.

### 2.2 Mouse model of GBM

Tumors were induced in 12 mice (9 males and 3 females), as previously described (Simões et al., 2008). Briefly, intracranial stereotactic injection of 1 x10^5^ GL261 or CT2A cells was performed in the caudate nucleus; analgesia (Meloxicam 1.0 mg Kg^-1^ s.c.) was administered 30 min before the procedure. Mice were anesthetized with isoflurane (1.5–2.0 % in air) and immobilized on a stereotactic holder (Kopf Instruments, Tujunga/CA, USA) where they were warmed on a heating pad at 37 °C, monitoring the body temperature with a rectal probe (WPI ATC-2000, Hitchin, UK). The head was shaved with a small trimmer, cleaned with Betadine, and the skull exposed through an anterior-posterior incision in the midline with a scalpel. A 1 mm hole was drilled in the skull using a micro-driller, 0.1 mm posterior to the bregma and 2.32 mm lateral to the midline. The tumor cells (1x10^5^ in 4 μL PBS) were inoculated 2.35 mm below the cortical surface using a 10 µL Hamilton syringe (Hamilton, Reno NV, USA) connected to an automatic push–pull microinjector (WPI *Smartouch^TM^*, Sarasota FL, USA), by advancing the 26 G needle 3.85 mm from the surface of the skull (∼1 mm skull-to-brain surface distance), pulling it back 0.5 mm, and injecting at 2 μL/min rate. The syringe was gently removed 2 min after the injection had finished, the skin sutured with surgical thread (5/0 braided silk, Ethicon, San Lorenzo Puerto Rico) and wiped with Betadine. The animals were kept at 25 °C during recovery from anesthesia, and given an opioid analgesic (Buprenorphine 0.05 mg Kg^-1^ s.c.) before returning to their cage. Meloxicam analgesia was repeatedly administrated at 24- and 48-hours post-surgery.

### 2.3 *In vivo* imaging of GL261 tumors

#### 2.3.1 Longitudinal MRI

GBM-bearing mice were imaged every 5–7 days on a 1 Tesla Icon MRI scanner (Bruker BioSpin, Ettlingen, Germany; running under *ParaVision 6.1*), to measure tumor volumes. For this, each mouse was placed in the animal holder under anesthesia (1–2 % isoflurane in 31 % O_2_), heated with a recirculating water blanket, and monitored for rectal temperature (36–37 °C) and breathing (60–90 BPM). Tumor volume was measure with T2-weighted ^1^H-MRI (*RARE* sequence, x8 acceleration factor, 2500 ms TR, 84 ms TE, NS 8, 1 mm slice thickness, and 160 µm in-plane resolution), acquired in two orientations (coronal and axial). Each session lasted up to 30 min/animal.

#### 2.3.2 End-point MRI/MRS

GBM-bearing mice were scanned 2–3 weeks post-induction on a 9.4 T BioSpec MRI scanner (Bruker BioSpin, Ettlingen, Germany; running under *ParaVision 6.1*), using a customized ^2^H/^1^H transmit-receive surface coilset (NeosBiotec, Pamplona, Spain) with a 11x15 mm inner loop (^2^H) and a butterfly configuration (^1^H), providing quadrature B_1_ field orientation to minimize coil coupling (adapted from a previous model for proton-decoupled ^13^C/^31^P spectroscopy (Adriany and Gruetter, 1997)). The coilset was initially tested *in vitro* (phantoms) to evaluate the sensitivity for dynamic and DGE ^2^H-MRS, and the performance of outer-volume suppression (OVS) for volume selection (**Supplementary Figs. 1 and 2**). Before each experiment, GBM-bearing mice fasted 4–6 h, were weighed, and cannulated in the tail vein with a catheter connected to a home-built 3-way injection system filled with: 6,6′–^2^H_2_-glucose (1.6 M in saline); Gd-DOTA (25 mM in saline); and with heparinized saline (10 U mL^-1^). Mice were placed on the animal holder under anesthesia (as in 2.3.1). Coilset quality factors (Q) for ^1^H and ^2^H channels were estimated in the scanner for each sample based on the ratio of the resonance frequency (61.45 and 400.34 MHz, respectively) to its bandwidth (full width at half-minimum of the wobbling curve during the initial tuning adjustments): 213 ± 3 and 168 ± 3, respectively. The mice were imaged first with T2-weighted ^1^H-MRI (*RARE* sequence, x8 acceleration factor, 3000 ms TR, 40 ms TE; 2 averages, 1 mm slice thickness, 70 µm in-plane resolution) in two orientations (coronal and axial). Then, the magnetic field homogeneity was optimized over the tumor region based on the water peak with ^1^H-MRS (*STEAM* localization: 28 ± 2 mm^3^ voxel size) using localized 1^st^ and 2^nd^ order shimming with the *MapShim Bruker* macro, leading to linewidths at half-maximum of 29 ± 3 Hz (**Supplementary Table 1**).

^2^H-MRS was performed using a *pulse-acquire* sequence, with 175 ms TR, 256 points, 1749 Hz, square *bp* RF pulse (0.128 ms, 10 kHz) with 55° flip angle, and outer volume suppression (OVS) with 6 pulses (10 mm slabs), to excite only the tumor region according to the previously defined *STEAM* voxel. After RF pulse calibration (using the natural abundance DHO peak), OVS-selective DGE ^2^H-MRS data were acquired for 90 min (32 k repetitions), with i.v. bolus of 6,6′–^2^H_2_-glucose (2 g Kg^-1^, injected over 30 s; Euroisotop, St Aubin Cedex, France).

Finally, animals underwent DCE T1-weighted ^1^H-MRI (*FLASH* sequence, 8° flip-angle, 16 ms TR, 4 averages, 150 repetitions, 3 slices with 1 mm thickness each, 140 µm resolution) with i.v. bolus injection of Gd-DOTA (0.1 mmol Kg^-1^, injected over 30 s; Guerbet, Villepinte, France). After MR examination, blood glucose was measured with a glucometer (OneTouch Select Plus Flex, LifeScan, Zug, Switzerland): 8.1 ± 1.0 mM, corresponding to euglycemia. Animals were then sacrificed under full anesthesia, their brains removed, washed in PBS, and immersed in 4 % PFA.

### 2.4 MRI/MRS processing

#### 2.4.1 T2-weighted ^1^H-MRI

T2-w MRI data were processed in ImageJ 1.53a (Rasband, W.S., ImageJ, U. S. National Institutes of Health, Bethesda, Maryland, USA, https://imagej.nih.gov/ij/, 1997–2018). For each animal, the tumor region was manually delineated on each slice, and the sum of the areas multiplied by the slice thickness to estimate the estimate the volume, which was averaged across the two orientations acquired (coronal and axial). In addition, the pixel intensities from each slice were normalized to a reference region (ROI in cortex, from the contra-lateral hemisphere), and the pixel distributions for each tumor analyzed for skewness, kurtosis, and inter-quartile range (IQR).

#### 2.4.2 DGE ^2^H-MRS

DGE ^2^H-MRS data were processed in MATLAB® R2018b (Natick, Massachusetts: The MathWorks Inc.) and jMRUI 6.0b (Stefan et al., 2009). Each dataset was averaged to 3 min temporal resolution (additional details in **Supplementary Methods**) and denoised with model-independent Marchenko–Pastur Principal Component Analysis (MP-PCA), taking the spectral and temporal dimensions as an M × N matrix and without any *a priori* constrains or assumptions, as originally reported (Veraart et al., 2016). Then, spectra were analyzed by individual peak fitting with AMARES (as before (Simões et al., 2015)), using a basis set for DHO (4.76 ppm: short- and long-T2 fractions (De Feyter et al., 2018)) and deuterium-labelled: glucose (Glc, 3.81 ppm), glutamine-glutamate (Glx, 2.36 ppm), and lactate (Lac, 1.31 ppm); relative linewidths referenced to the estimated short-T2 fraction of DHO, according to the respective T2 relaxation times reported by de Feyter *et al* (De Feyter et al., 2018). The natural abundance DHO peak (DHO_i_) was further used to select and quantify both original and denoised spectra: SNR_DHOi_ > 15 and 13.88 mM reference (assuming 80 % water content in the brain and 0.03 % natural abundance of DHO), respectively. Metabolite concentrations (CRLB < 50 %; otherwise discarded) were then corrected for T1 and labeling-loss effects, according to the values reported by de Feyter *et al* (T1, ms: DHO, 320; Glc, 64; Glx, 146; Lac, 297) (De Feyter et al., 2018) and de Graaf *et al* (number of magnetically equivalent deuterons: DHO, 1; Glc, 2; Glx, 1.2; Lac, 1.7) (de Graaf et al., 2021), respectively. Thus, the concentration of each metabolite (m) at each time point was estimated as (Eq (1)):

(1) $${\mathrm{Conc}}_{m}=\frac{{\mathrm{Area}}_{m}-{Area0}_{m}}{d_{m}}\times \frac{C_{\mathrm{DHO}}}{C_{m}}\times \frac{d_{\mathrm{DHO}}}{{Area0}_{\mathrm{DHO}}}\times {\mathrm{Conc}}_{\mathrm{ref}}$$

*Area* = peak area; *Area0* = average peak area before injection; *d* = number of magnetically equivalent deuterons corrected for labelling-loss effects; *C* = T1 correction factor (1-exp(-TR/T1)); and *Conc_ref_* = reference DHO concentration.

The time-course changes of ^2^H-labelled metabolite (Glc, Glx and Lac) concentrations were fitted using a modified version of the kinetic model reported by Kreis et al (Kreis et al., 2020), to estimate the maximum rate of Glc consumption (total, *V_max_*) for Glx synthesis (mitochondrial oxidation, *V_glx_*) and Lac synthesis (glycolysis, *V_lac_*), and the confidence intervals for all estimated parameters:

(2) $$V_{\max}=V_{\mathrm{lac}}+V_{\mathrm{glx}}$$

The coupled differential equations describing the concentration kinetics of each metabolite were:

(3) $$\frac{d\left[Glc\right]}{\mathrm{dt}}\;=\;k_{g\;}\left(C_{p}-\frac{\left[Glc\right]}{v}\right)\;-\;f\;\left(\frac{V_{\max}\;C_{p}}{f\;v\;k_{m}+C_{p}}\right)$$

(4) $$\frac{d\left[Lac\right]}{\mathrm{dt}}=\frac{f\;V_{\mathrm{lac}}\;C_{p}}{f\;v\;k_{m}+C_{p}}\;-\;k_{l}\;\left[Lac\right]$$

(5) $$\frac{d\left[Glx\right]}{\mathrm{dt}}=\frac{f\;V_{\mathrm{glx}}\;C_{p}}{f\;v\;k_{m}+C_{p}}\;-\;K_{l}\;\left[Glx\right]$$

where: *k_g_*, apparent rate constant of glucose transfer between blood and tumor (min^−1^); *k_glx_*, apparent rate constant of Glx elimination (min^−1^); *k_lac_*, apparent rate constant of lactate elimination (min^−1^); $C_{p}=a_{1}e^{-k_{p}t},$ Glc concentration in plasma (mM); *a_1_*, the Glc concentration after the bolus injection (mM); *k_p_*, the effective rate constant of labeled glucose transfer to tissue (min^−1^); *f*, the fraction of deuterium enrichment; *v*, the extravascular-extracellular volume fraction; and *k_m_*, the constant for glucose uptake. As originally reported (Kreis et al., 2020), the fraction of deuterium enrichment (*f*) and constant for glucose uptake (*k_m_*) were fixed: the former, using the same estimation (*f* = 0.6, based on NMR of blood samples) since the injection protocol and dose/weight used were the same and also in mice; the latter, approximated to *k_m_* = 10 mM (Marín-Hernández et al., 2011, Williams et al., 2012). All parameters were fitted without any restrictions to their range.

#### 2.4.3 DCE T1-weighted ^1^H-MRI

DCE T1-w MRI data were processed with DCE@urLab (Ortuno et al., 2013). ROIs were manually delineated for each slice and the time-course data was fitted with the Extended Tofts 2-compartment model (Tofts, 1997), to derive the volume transfer constant between plasma and tumor extravascular-extracellular space (*K^trans^*), the washout rate between extravascular-extracellular space and plasma (*k^ep^*), and the extravascular-extracellular volume fraction (*v*). The measurements were averaged across 3 slices for each tumor, covering the whole lesion, and were consistent with the literature for mouse brain tumors, e.g. *K^trans^* (Boult et al., 2017).

### 2.5 Metabolic assessment *in situ*

GL261 and CT2A cells were seeded overnight on Seahorse XFp miniplates (1x10^4^ cells well^-1^). After attachment/growth for 18 h, the RPMI_compl_ medium in each well was changed to unbuffered medium (103575-100, Agilent, Santa Clara CA, USA) with the same glutamine and glucose concentration as in RPMI_compl_ and incubated for 1 h in a CO_2_-free atmosphere. The cells were then studied *in situ* using a Seahorse XF HS Mini Analyzer (Agilent, Santa Clara CA, USA) using the *MitoStress Test Kit.* The *MitoStress Test Kit* measures the extracellular acidification rate (ECAR) and the oxygen consumption rate (OCR) in each well while specific inhibitors of the respiratory chain are sequentially injected. The data were processed with Seahorse Analytics (Agilent, Santa Clara CA, USA) and normalized to the cell number for each well. The latter were determined at the end of the experiment from the cell lysates (RIPA buffer, prepared in-house) using the bicinchoninic acid protein assay (Pierce^TM^ BCA Protein kit, ThermoFisher Scientific, Rockford IL, USA) and a microplate reader (BioTek ELMX800, Cole-Parmer, Winooski VT, USA), and assuming a total protein content 2500 µg L^-1^ x10^7^ cells, as before (Simões et al., 2015).

### 2.6 Histopathology and immunohistochemistry

Whole brain was fixed in 4 % PFA, embedded in paraffin and serially sectioned at 4 μm in 30 different levels in its horizontal plane, spanning the whole tumor area. Sections were stained with H&E (Sigma-Aldrich, St. Louis MO, USA) and analyzed by a pathologist blinded to experimental groups. Tumor proliferation index was assessed in sections immunostained for Ki67 (mouse anti-ki67, BD, San Jose CA, USA; blocking reagent, M.O.M ImmPRESS kit, Vector Laboratories, Burlingame CA, USA; liquid DAB^+^, Dako North America Inc, Carpinteria CA, USA): 6–9 sections were quantified for each sample, representative of the entire tumor volume. The latter were digitized (Nanozoomer, Hamamatsu, Japan) and analyzed with QuPath 0.2.3 (https://qupath.readthedocs.io/en/latest/), blindly from DGE ^2^H-MRS results. Thus, the tumor regions on each slide were manually defined with ROIs, followed by semi-automated counting of Ki67^+/-^ cells to determine the total cell density and the labeling index (% Ki67^+^ cells). Finally, the total cell number for each tumor was estimated based on the total tumor volume (T2-w MRI data), the average cell count per surface area (histologic counting) and assuming a cell radius of 10 µm (as reported in mouse GL261 tumors (Roberts et al., 2020)).

### 2.7 Statistical analyses

Data were analyzed using the two-tailed Student’s *t-*test. Differences at the 95 % confidence level (p = 0.05) were considered statistically significant. Correlation analyses were carried out with the Pearson R coefficient, unless indicated otherwise.

## 3 Results

### 3.1 MRI assessment of GBM allograft tumors

Orthotopic GL261 and CT2A tumors were studied *in vivo* 19 ± 1 days post-injection, during a well-described epoch of active cell proliferation before marked necrosis (Cha et al., 2003, Martinez-Murillo and Martinez, 2007, Zagzag et al., 2000). Volumetric (T2-w) and DCE T1-w ^1^H-MRI of the entire cohort showed consistent tumor sizes (38.3 ± 3.4 mm^3^) and perfusion properties, respectively (**Supplementary Table 1** and Fig. 3), in agreement with previous studies (Cha et al., 2003). Additional analysis revealed similar heterogeneity based on T2-w MRI contrast in both GBM models (skewness, kurtosis and inter-quartile range assessment of pixel distributions) but significantly higher magnetic field homogeneity achieved in CT2A tumors (-34 % linewidth of ^1^H-MRS-detectable tumor water, p = 0.011; leading to higher sensitivity for ^2^H-MRS detection, +23 % signal-to-noise ratio of the original data, p = 0.019 – **Supplementary Table 1**), which suggested more heterogeneous microenvironments in GL261 tumors.

### 3.2 Quantification of glycolytic and oxidative consumption of glucose in GBM tumors *in vivo*

**Fig. 2:**
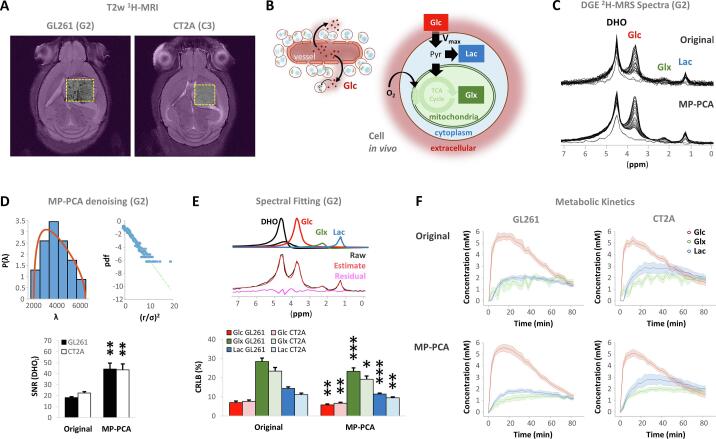
**DGE ^2^H-MRS of mouse GL261 and CT2A tumors *in vivo*. A** T2-w MRI of GL261 (G2) and CT2A (C3) tumors, displaying the DGE ^2^H-MRS volume of interest (yellow dashed line) and OVS regions (purple). **B** Metabolic model: Glc extravasation, cell uptake and maximum consumption rate (*V_max_*) through glycolysis (Lac synthesis, *V_lac_*) and mitochondrial oxidation via the TCA cycle (Glx synthesis, *V_glx_*). **C** Stacked DGE ^2^H-MRS data before and after denoising (original and MP-PCA, respectively), with peak assignments. **D** MP-PCA denoising: left-side top, eigenvalue spectrum from PCA decomposition (blue) and fit of MP distribution (orange); right-side top, Gaussian distribution of denoising residuals, verified by the linearity of their logarithm; bottom, signal-to-noise ratio of the basal DHO peak (SNR_DHOi_) before and after denoising. **E** Spectral fitting: top, time-domain fitting of MP-PCA spectra for absolute quantification (top - components; bottom - black, original; red, estimate; magenta, residuals), and kinetic fitting for Glc (red), Glx (green) and Lac (blue); bottom, Cramer-Rao lower bounds of the spectral fits in the original data and after MP-PCA denoising (Glc, first 10 spectra after bolus injection; Glx and Lac, last 10 spectra). **F** Time-course profiles of metabolic concentrations in the original data and after MP-PCA denoising, which improved Glx detection. DHO, semi-deuterated water; Glc, 6,6′-^2^H_2_-glucose; Glx, 4,4′-^2^H-glutamate-glutamine; Lac, 3,3′-^2^H-lactate; TCA, tricarboxylic acid. Plots: mean ± SE, n = 7 GL261 and n = 5 CT2A. * p < 0.05, ** p < 0.01, *** p < 0.001.

We then performed localized DGE ^2^H-MRS (Fig. 2**A**) to monitor the deuterium-labelled glucose (Glc) dynamics; namely, conversion to its downstream products lactate (Lac) and glutamate-glutamine pool (Glx) (Fig. 2**B**). The location of the voxel (shown in Fig. 2**A** for representative GL261 and CT2A glioma-bearing mice) was chosen such that it encompassed as much tumor volume as possible while avoiding peritumoral regions. The quality of the original spectra (Fig. 2**C** upper panel) reveals good spectral resolution and the ability to distinguish between the different ^2^H-labelled metabolite pools. The signal-to-noise ratio for the natural abundance DHO peak (SNRi) in the basal original spectra was 20.0 ± 0.9. To improve the detection limits and kinetic profiling, we harnessed a MP-PCA denoising strategy which clearly improved the spectral quality (Fig. 2**C**, lower panel). Specifically, MP-PCA indeed improved the SNRi to 43.9 ± 3.7, demonstrating a 2-fold gain (p < 0.001 in each tumor cohort) (Fig. 2**D**); and an average 19 ± 1 % improvement in spectral fitting precision (Fig. 2**E**) was noted. Specifically, MP-PCA denoising consistently improved the time-course detection of Glx concentration changes without altering the kinetics (Fig. 2**F**).

**Fig. 3:**
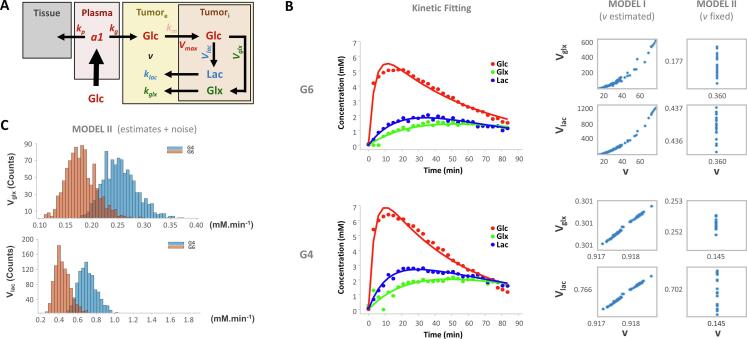
**Kinetic model for DGE ^2^H-MRS. A** Modification of the model proposed by Kreis et al. (Kreis et al., 2020) for measuring the maximum rate of glucose consumption (*V_max_*) for synthesis of lactate (*V_lac_*) and glutamate-glutamine (*V_glx_*): *V_max_* = *V_glx_* + *V_lac_*. **B** Performance of *V_glx_* and *V_lac_* estimates (mM min^−1^) when *v* (0–1) is taken as a model-free parameter of the kinetic model (model I) vs. when parameter *v* is fixed to the value obtained from DCE T1-w data (model II), demonstrated in two tumors: top, G4; bottom, G6. Different *V_glx_* and *V_lac_* estimates were obtained by running the fitting procedures with different initial guess estimates, which were uniformly sampled between maximum/minimum values of confidence interval range across all the samples. While for some samples model I and II estimates showed comparable results (G4: 0.301 and 0.252 for *V_glx_*, and 0.766 vs 0.702 for *V_lac_*, respectively), in other samples meaningful estimates were only obtained by fixing *v* to the value obtained from DCE T1-w data (G6: 0–600 and 0.117 for *V_glx_*, and; 0–1200 and 0.437 for *V_lac_*, respectively). **C** Precision of the model demonstrated by iteratively (x1000) adding random noise (1 %) to the parameter estimations obtained with *v* fixed (model II) and repeating the estimations with the same model at each step. While noisy estimates from different iterations render normal distributions, their values are around the initial estimates and consistently suggesting higher *V_glx_* and *V_lac_* for G4 compared to G6 (0.252 vs. 0.177, and 0.702 vs. 0.437, respectively). ***a_1_***, Glc concentration after the bolus injection (mM); ***k_p_***, effective rate constant of labeled glucose transfer to tissue (min^−1^); ***k_g_***, apparent rate constant of glucose transfer between blood and tumor (min^−1^); ***V_glx_***, maximum rate of Glc consumption for Glx synthesis (mM min^−1^); ***k_glx_***, apparent rate constant of Glx elimination (min^−1^); ***V_lac_***, maximum rate of Glc consumption for Lac synthesis (mM min^−1^); ***k_lac_***, apparent rate constant of lactate elimination (min^−1^); ***V_max_***, maximum rate of total Glc consumption (mM min^−1^).

Adaptation of a previous kinetic model (Kreis et al., 2020) enabled the estimation of maximum glucose consumption rates (*V_max_*) in GL261 tumors *in vivo*, for synthesis of Lac – glycolysis (*V_lac_*) – and Glx – mitochondrial oxidation (*V_glx_*) (Fig. 3**A)**. This was initially tested in parallel with two variants of the model, including the extracellular volume fraction (*v*) either as a variable (*v estimated*) or fixed according to its estimation from the respective DCE T1-w MRI experiment (*v fixed*), using the Extended Tofts Model (Supplementary Fig. 3). While most samples (e.g. G4) consistently rendered comparable *V_glx_* and *V_lac_* estimates taking *v* as either a model-free or model-fixed parameter, other samples (e.g. G6) did not (Fig. 3**B**). Specifically, *v estimated* and *v fixed* models mostly converged to very close estimates of *V_lac_* and *V_glx_* (respectively fold-changes: 1.2 ± 0.1 (p = 0.140) and 1.1 ± 0.1 (p = 0.151), for GL261 (G1, G2, G3, G4, G5 and G7); and 1.2 ± 0.1 (p = 0.373) and 0.9 ± 0.0 (p = 0.374), for CT2A (C1,C2,C3,C4)). However, this was not verified in samples G6 and C5: while the *v fixed* model also converged to *V_lac_* and *V_glx_* estimates within the expected physiologic ranges, the *v estimated* model drifted to very large *V_lac_* and *v* estimates without biological meaning, as demonstrated for the G6 sample (Fig. 3**B**). Therefore, the *v fixed* approached enabled the “stabilization” of the model for all the samples in each cohort. The precision of *V_glx_* and *V_lac_* estimations with the *v fixed* model was also demonstrated (Fig. 3**C)**.

### 3.3 Heterogeneity of glucose metabolism in GBM tumors *in vivo*

**Fig. 4:**
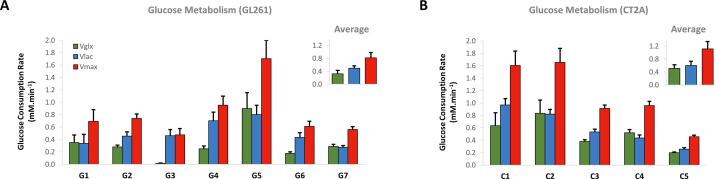
**Glucose consumption rates in mouse GBM tumors**. Estimated maximum rates of glucose consumption in GL261 (**A**) and CT2A (**B**) tumors, for *de novo* synthesis of (estimate ± SE mM min^−1^): Glx, 0.40 ± 0.08 (*V_glx_*, green); Lac, 0.54 ± 0.07 (*V_lac_*, blue); and total, 0.94 ± 0.13 (*V_max_*, red).

Harnessing the kinetic model with extracellular volume fixed to that measured from DCE, the estimations of *V_lac_* (0.54 ± 0.07 mM min^−1^) and *V_glx_* (0.40 ± 0.08 mM min^−1^) in GBM allograft tumors could be robustly extracted (Fig. 3, Fig. 4). Marked inter-tumor metabolic heterogeneity in glucose consumption rate through glycolysis and mitochondrial oxidation, respectively, was clearly demonstrated (Fig. 4). Thus, while some tumors appeared to rely mostly on glycolytic turnover of glucose (e.g. G3, V_lac_ = 0.46 ± 0.1 and V_glx_ = 0.02 ± 0.01 mM min^−1^), other tumors ranged up to similar glucose consumption rates through each pathway flux (e.g. C4, V_lac_ = 0.44 ± 0.5 and V_glx_ = 0.52 ± 0.05 mM min^−1^;).

### 3.4 Functional assessment of mitochondrial respiration and glycolysis in glioma cell lines *in situ*

**Fig. 5:**
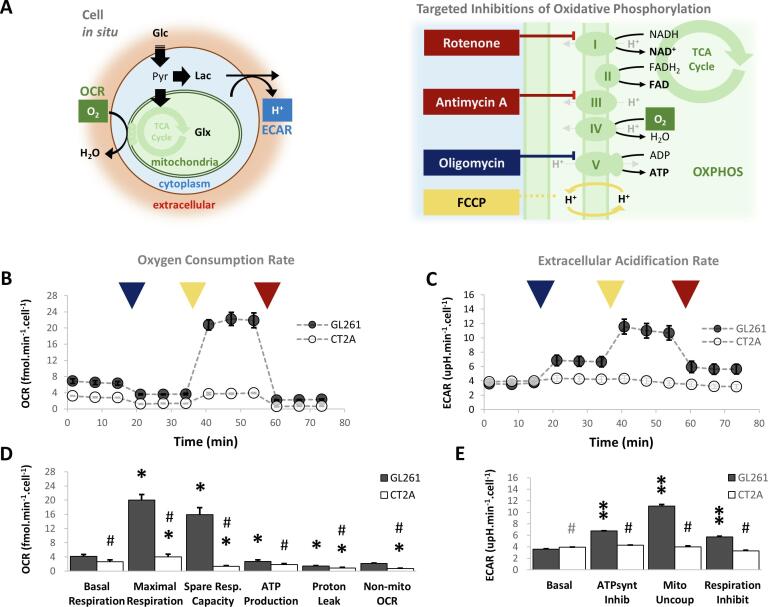
**Functional metabolic assessment of GL261 and CT2A cells *in situ*. A** Extracellular flux analysis of oxygen consumption rate (OCR) and extracellular acidification rate (ECAR) in glioma cells proliferating on micro-well plates during stepwise inhibition of OXPHOS: mitochondrial ATP synthase by oligomycin (dark blue), mitochondrial uncoupling by FCCP (yellow), and complex I by rotenone/antimycin-A (dark red). **B-E** Time-course monitoring of OCR (**B**) and ECAR (**C**) and respective parameters derived for mitochondrial OXPHOS (**D**) and glycolysis (**E**). Average cell count estimates in each well (x10^-3^ cells µm^−2^): GL261, 3.44 ± 1.49; CT2A, 5.50 ± 1.38. Plots: mean ± SE (n = 3); * p < 0.05, ** p < 0.01 vs. basal condition; # p < 0.001 (grey p < 0.05) vs. GL261.

To investigate whether our *in vivo* findings were related to basal metabolic properties of each tumor model, additional *in situ* analysis were performed for functional metabolic assessment of the respective cell lines. Specifically, we investigated glycolysis and mitochondrial oxidation metabolism at the cellular level during active proliferation in 2D cell culture (Fig. 5**A**). The results clearly indicated simultaneous mitochondrial OXPHOS (Fig. 5**B**) and glycolytic metabolism (Fig. 5**C**) under regular growth conditions (basal respiration, Fig. 5**D-E**) in GL261 and CT2A cells – namely, 4.13 ± 0.52 and 2.67 ± 0.49 fmol O_2_ min^−1^ cell^−1^ OCR (respectively), within the range of literature values reported with primary GBM cells and other GBM cell lines (Arthurs et al., 2020). While the results indicate oxygen consumption mostly coupled to ATP production in both cell lines (>65 % of basal respiration, Fig. 5**D**), GL261 and CT2A cells revealed marked differences in mitochondrial function. Thus, GL261 cells had a significant respiration buffer (maximal and spare respiration capacity ∼ 5-fold higher than basal respiration, Fig. 5**D**), not observed in CT2A cells. In addition, only GL261 cells demonstrated precise increases of glycolytic flux during stepwise inhibition of OXPHOS (Fig. 5**E** – basal ECAR measurements also within the range of previous studies in primary GBM cells and other GBM cell lines (Arthurs et al., 2020)), revealing an efficient metabolic plasticity.

### 3.5 Histopathology of GBM tumors

**Fig. 6:**
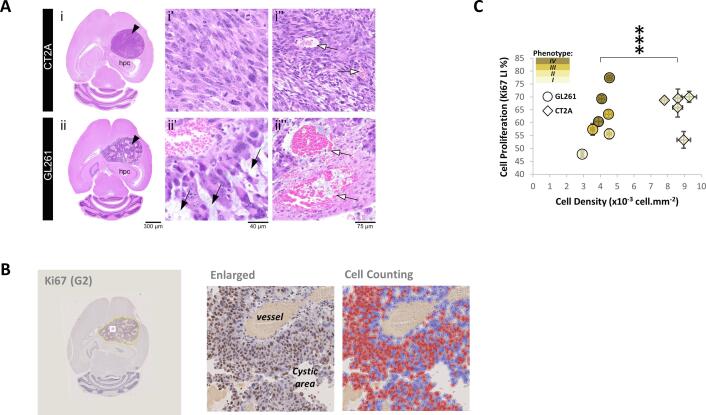
**Histopathologic analyses of GBM tumors. A** Representative microphotographs of H&E-stained CT2A (I, C3) and GL261 (ii, G2) brain tumors, showing well circumscribed lesions (black arrowhead) of similar size, in the same anatomical location, adjacent to and compressing the hippocampus (hpc). The tumors show distinct morphological features: CT2A are composed by dense, cohesive and homogeneous tumor cell population (i’), rich in small vessels with preserved integrity (white arrow) (ii’’); while GL261 tumors show marked heterogeneity, with poorly cohesive areas and marked intercellular edema (black arrow) (ii), that intercalate with large dilated vessels, occasionally with loss of endothelial lining (white arrowhead) and extensive hemorrhages (ii). The various phenotypes encountered in the stromal-vascular fractions of these tumors are described in detail in **Supplementary**Fig. 4. **C** Ki67 immunostaining of a brain section (G2) displaying the tumor ROI for quantification (yellow-line), with enlarged view of a sub-tumor region (white-rectangle) showing Ki67^+^ (brown) and Ki67^-^ (blue) cells, and respective counting (mean cell densities: GL261, 4.0 ± 0.2; CT2A, 8.6 ± 0.2 x10^-3^cells µm^−2^). **D** Cell detection measurements in 7.0 ± 0.4 histologic sections for each tumor (means ± SE displayed), showing significantly higher density in CT2A tumor within the same proliferation range. Legend: black-arrow, small vessels; white-arrow, vasodilation; asterisk, multifocal hemorrhages; black-arrowhead, incomplete endothelial cell lining; cardinal, edematous stroma white-arrowhead, edematous stroma. *** p < 0.0001.

Given that our *in situ* findings could suggest differential metabolic adaptability of GL261 and CT2A cells to microenvironmental changes during GBM proliferation, potentially consistent with the heterogeneity of glucose metabolic fluxes detected *in vivo* (Fig. 4), histopathological analysis was performed. Tumors were screened for cytomorphological features, cell density, presence of hemorrhage, necrosis, and for cell proliferation index, blinded to *in vivo* MRI/MRS data (Fig. 6). While no significant difference was seen in extent of necrosis between GL261 and CT2A tumors, only the latter displayed extensive morphologic heterogeneity (Fig. 6**A**, **Supplementary Table 2**), in agreement with the differences in magnetic field homogeneity detected *in vivo* in each model (**Supplementary Table 1**). Specifically, GL261 tumors showed distinct morphological features associated with progressive compression by the expansile tumor growth, including compression of vasculature, damage to the vessels, hemorrhage and edema. Tumors were scored individually for the following stromal-vascular phenotype: I, small vessels, complete endothelial cell lining and sparse hemorrhages; II, vasodilation and marked multifocal hemorrhages; III, necrosis of the vascular wall, incomplete endothelial cell lining, vascular leakage, and edematous stroma; and IV, vascular depletion and edematous stroma (Supplementary Fig. 4). Further assessment of cell proliferation index based on ki67 immunostaining (Fig. 6**B**) indicated a strong correlation of this parameter with the stromal-vascular fraction scores in GL261 tumors (Kendall’s rank coefficient: τ = 0.80, p = 0.017), which was not observed in CT2A tumors (Fig. 6**C**). The latter displayed 2-fold higher cellularity than GL261 tumors (Fig. 6**C**; 8.7 ± 0.2 vs 4.0 ± 0.2 x10^8^ cells mL^−1^, p < 0.0001 – **Supplementary Table 1**) and a remarkable homogeneity of cell morphology, with small vessels, complete endothelial cell lining and sparse hemorrhages, consistent with a stromal-vascular phenotype I (**Supplementary Table 2**).

### 3.6 Modulation of glucose metabolism according to cell proliferation in GBM tumors

**Fig. 7:**
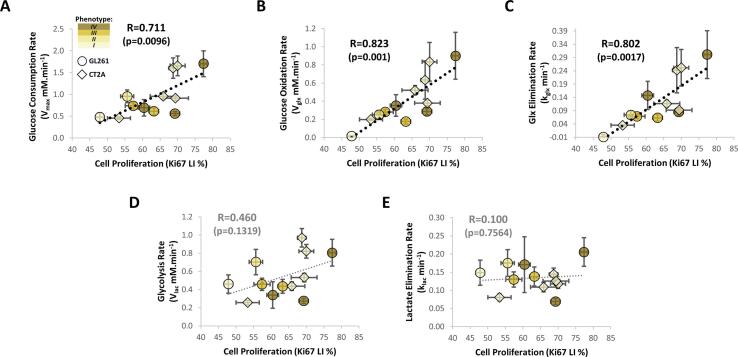
**Association between glucose metabolism and cell proliferation in pooled GL261 and CT2A tumor cohorts.** Cell proliferation index correlated significantly with glucose consumption rate (**A**), which was associated with its mitochondrial oxidation, i.e. rates of Glx synthesis (**B**) and elimination (**C**), rather than glycolytic turnover, i.e. rates of Lac synthesis (**D**) or elimination (**E**). Plots: estimate ± CI for glucose metabolism-derived metrics, and means ± SE for cell proliferation index.

Finally, we explored potential associations between glucose metabolic heterogeneity and tumor microenvironment features in GBM allografts (Fig. 7**A**). Thus, glucose consumption rate correlated significantly with cell proliferation index across the pooled cohorts (n = 12) and regardless of the histopathologic phenotype (R = 0.71, p = 0.010 – Fig. 7**A**). Importantly, this was associated specifically with glucose mitochondrial oxidation (i.e. Glx synthesis and elimination rates: R = 0.82, p = 0.001; and R = 0.80, p = 0.002, respectively – Fig. 7**-B** and **7-C**) rather than glycolysis (Fig. 7**D** and **E**), which was already detectable in the more heterogeneous GL261 model (R = 0.84, p = 0.018; and R = 0.84, p = 0.019, respectively – **Supplementary Table 1**). Moreover, no association was observed between cell proliferation and other tumor parameters, such as volume, perfusion, heterogeneity based on T2-w MRI contrast, histopathologic phenotype, or cell density (**Supplementary Table 1**).

## 4 Discussion

The ability of DGE ^2^H-MRS to quantify glucose metabolic rates through mitochondrial oxidation (normal rat brain (Lu et al., 2017)) or glycolysis (subcutaneous mouse tumors (Kreis et al., 2020)), has been rapidly gaining interest for *in vivo* metabolic flux assessment. Despite the great promise for *in vivo* DGE ^2^H-MRS, the typically low signal-to-noise ratio in these experiments, which is mainly incurred due to deuterium’s low resonance frequency (compared with its ^1^H counterpart), imposes significant boundaries on their ability to characterize the relevant metabolic rates and/or accurately define relatively weak signals, such as Glx. Thus, simultaneous characterization of both glycolytic and oxidative pathways remains limited. This work aimed to harness the DGE ^2^H-MRS methodology with volume selection and MP-PCA spectral denoising to overcome these major limitations, and investigate whether such simultaneous measurements could address the current need to detect specific metabolic pathway-dependencies in GBM tumors (Garofano et al., 2021). Furthermore, we investigated the potential relevance of DGE ^2^H-MRS for characterizing the links between metabolic heterogeneity and histopathologic features, including cell proliferation.

To test these features, we used two robust, immunocompetent mouse models mimicking clinical GBM – GL261 and CT2A (Griguer et al., 2005, Martinez-Murillo and Martinez, 2007, Oh et al., 2014, Seligman and Shear, 1939, Seyfried et al., 1992, Zagzag et al., 2000) – which evidenced pronounced histologic and metabolic heterogeneity and thereby were suitable for testing our hypotheses. Our three-pronged strategy was designed to: (i) measure the *in vivo* glucose metabolic rates in orthotopic GBM tumors using DGE ^2^H-MRS and a novel unbiased denoising strategy (MP-PCA (Veraart et al., 2016, Does et al., 2019)) that increases the confidence and robustness of the fits; (ii) contrast these metrics with functional metabolic assessment of the respective cell lines under controlled *in situ* conditions; and (iii) assessing the tumor heterogeneity via unbiased histopathologic and cell proliferation assessment.

The *in vivo* DGE ^2^H-MRS-driven estimates of glycolytic flux in GL261 tumors (*V_lac_*: GL261, 0.50 ± 0.07 mM min^−1^ or 1.25 ± 0.16 fmol min^−1^ cell^−1^; CT2A, 0.60 ± 0.13 mM min^−1^ or 0.70 ± 0.16 fmol⋅min^−1^⋅cell^−1^) were within the ranges reported in the literature for other tumor models, such as: mouse lymphoma, also using *in vivo* DGE ^2^H-MRS (0.99 mM min^−1^ (Kreis et al., 2020)); rat breast cancer xenografts, based on biochemical assay analysis of tissue-isolated samples (1.43 mM min^−1^ (Kallinowski et al., 1988), assuming 1 mL ∼ 1.1 g); and even perfused U87 GBM cells, using *in situ* hyperpolarized ^13^C-MRS (∼1.35 fmol min^−1^ cell^−1^ (Jeong et al., 2017)). With regards to glucose consumption rate through mitochondrial oxidation (*V_glx_*: GL261, 0.32 ± 0.10 mM min^−1^ or 0.77 ± 0.23 fmol min^−1^ cell^−1^; CT2A, 0.51 ± 0.11 mM min^−1^ or 0.60 ± 0.12 fmol min^−1^ cell^−1^), our estimates were closely consistent with the *in situ* results in cell culture (up to 0.69 ± 0.09 and 0.44 ± 0.08 fmol min^−1^ cell^−1^, respectively – assuming a 1 Glc : 6 O_2_ stoichiometry to fully sustain OXPHOS), thereby validating the robustness of these *in vivo* measurements towards estimating glucose metabolic fluxes. Moreover, although the kinetic model used is rather simplistic compared to more established ones for assessing cerebral metabolic rates of glucose consumption in healthy rodent brain with labelled tracers, including deuterated glucose (0.25 mM min^−1^ (Lu et al., 2017)), our findings are also well aligned with prior ^13^C-MRS-based estimates in mouse GBM xenografts (0.33 mM min^−1^ (Lai et al., 2018)), lending further credence to this strategy.

Assessment of mitochondrial function *in situ* further suggested the coupling between basal respiration and energy production in both glioma cell lines. While this should be taken with caution considering glutamine-driven mitochondrial substrate level phosphorylation in the glutaminolysis pathway for ATP synthesis in several cancer cells, including CT2A (Chinopoulos and Seyfried, 2018, Duraj et al., 2021), both approaches link ATP production to mitochondrial metabolism. More importantly, there were marked metabolic differences between the two cell lines, consistent with different sensitivities to treatment previously reported (McKelvey et al., 2021), and distinct histopathologic features of the respective tumors. GL261 cells demonstrated strong respiration buffer capacity (up to 5-times the basal respiration rate) and efficient metabolic plasticity between glycolysis and mitochondrial oxidation, generating orthotopic tumors with heterogeneous stromal-vascular phenotypes (scores I-IV) closely reflecting their cell proliferation index, which in turn correlated strongly with glucose mitochondrial oxidation *in vivo*. Thus, despite the instability and disruption of tumor-associated vessels toward more aggressive histopathologic phenotypes, the overall good perfusion of non-necrotic GL261 tumors *in vivo* should support the oxygen demands for increasing mitochondrial metabolism, likely to sustain the high anabolic and energetic requirements for cell proliferation while protecting against reactive oxygen species in the hemorrhagic stroma (Weinberg et al., 2019).

Interestingly, while the glucose oxidation fraction (*V_glx_*/*V_max_*) was significantly higher in GL261 tumors with more aggressive phenotype (IV vs I-II-III, p = 0.026) under regular anesthesia conditions (fraction of inspired oxygen (FiO_2_), 31 %; blood oxygen saturation (SpO_2_), 98.8 ± 0.2 %), preliminary data with additional GL261 tumors indicated decreased glucose oxidation fraction under acute hypoxia (FiO_2_, 12 %; SpO_2_, 61.9 ± 1.6 % – Supplementary Fig. 5 **and Supplementary Tables 2 and 3**). This was consistent with the metabolic plasticity demonstrated *in situ* during OXPHOS inhibition and suggests quick metabolic adaptation to lower oxygen tensions, typically present in advanced malignant tumors. Accordingly, metabolic plasticity represents a key element for cancer cell survival, adaptation, and proliferation in a rapidly shifting microenvironment (Fendt et al., 2020, Gillies et al., 2012, Lehuédé et al., 2016), with a pivotal role for mitochondrial reprogramming between e.g. invasive and proliferative states (the latter supported by oxidative metabolism (Li et al., 2020)), which are present in GBM (Rajapakse et al., 2020, Xie et al., 2014). Altogether, our observations strengthen the relevance of DGE ^2^H-MRS for *in vivo* detection of GBM dependencies on oxidative metabolism at any given progression stage. This could be helpful for early treatment assessment, by evaluating the response to: antiangiogenic therapies, which impact tumor perfusion (oxygenation) and therefore the ability to rely on oxidative metabolism (Batchelor et al., 2014); OXPHOS-targeted treatments (Molina et al., 2018, Shi et al., 2019); or even the efficiency of chemosensitization to those or other therapies, e.g. with dichloroacetate (Michelakis et al., 2010, Shen et al., 2015).

Compared to the GL261 model, CT2A cells demonstrated more limited metabolic flexibility *in situ*. Namely, CT2A cells displayed markedly reduced respiration buffer capacity and no apparent glycolytic response to acute inhibition of OXPHOS. This was consistent with a conserved, less aggressive stromal-vascular phenotype (score I) of CT2A tumors, and 2-fold higher cellular density than GL261′s. Despite such metabolic and histopathologic differences between the two allograft models, pooling them strengthen our previous finding that non-necrotic GBM tumors with consistent sizes and perfusion properties had an increasing reliance on glucose mitochondrial oxidation according to cell proliferation index. This is aligned with recent observations of increasing OXPHOS-dependance at more advanced stages of tumor progression (Faubert et al., 2020), and even polarization of tumor-associated macrophages towards an OXPHOS-dependent, pro-tumorigenic M2 phenotype (Van den Bossche et al., 2017). Thus, our results demonstrate the potential of DGE ^2^H-MRS for non-invasive detection of clinically relevant GBM phenotypes.

As in every study, we acknowledge the limitations of this work. Firstly, although the Glx peak region was assigned to the glutamate-glutamine pool, this region overlaps with other TCA-cycle intermediates/derivatives such as succinate (2.39 ppm), which has been detected e.g. in breast cancer cell lines with TCA-cycle truncations (Simões et al., 2015). While no such truncations have been reported in the GL261 model, and are not expected according to the *in situ* Seahorse experiments performed, the CT2A model could potentially harbor them given the electron transport chain abnormalities reported (Kiebish et al., 2008). In any case, the Glx region assigned in DGE ^2^H-MRS should still reflect *de novo* mitochondrial turnover of glucose. Secondly, the normal variations in tumor shape within each cohort led to some discrepancies in the DGE 2H-MRS voxel vs. total tumor volumes. Specifically, tumors G4 and C4 – the smallest in their respective cohorts – presented more regular shapes that mostly fitted the total voxel volume. While this led to ∼ 20 % larger voxel volume than total volume, the same tumor/non-tumor proportion was kept during visual adjustment of the voxel in these tumors (as in all others); otherwise, lower rates of glycolysis vs glucose oxidation would be expected in these samples due to stronger contaminations from non-tumor tissue, which was not the case (**Supplementary Table 1**, Vlac/Vglx: G4 > G2 ∼ G1 > G7 ∼ G5; C4 ∼ C1 > C2). Moreover, the differences in voxel vs tumor volumes did not correlate significantly to glucose oxidation or glycolytic rates within each cohort or across pooled cohorts (**Supplementary Table 4**), further suggesting no association between those variables, we performed the study at 9.4 Tesla, which represents a higher magnetic field strength compared to standard clinical scanners (1.5 to 3.0 Tesla). However, since deuterium spectroscopy and imaging (De Feyter et al., 2018) benefit significantly from increased field strengths, their translational application to the human brain has already been demonstrated at 7.0 Tesla (de Graaf et al., 2020) and could be extended to 9.4 Tesla human scanners.

## 5 Conclusion

This study demonstrated (i) the potential of DGE ^2^H-MRS for *in vivo* assessment of glucose consumption rates through glycolytic and oxidative pathways simultaneously in mouse GBM, and potentially other aggressive tumors with OXPHOS dependencies (Méndez-Lucas et al., 2020); and (ii) its relevance for metabolic characterization of GBM, as evidenced here by the strong association between the heterogeneity of glucose consumption rates and cell proliferation in two well-established allograft models prior to marked necrosis. The relevance of this novel application for non-invasive stratification and early assessment of treatment efficacy in GBM patients (Garofano et al., 2021) warrants its extension to additional tumor models and progression stages, and even patients. Given that clinical translation of deuterium imaging has already been demonstrated for GBM (De Feyter et al., 2018), and treatment-response monitoring with DGE deuterium spectroscopy and imaging reported in a mouse lymphoma model (Kreis et al., 2020), our findings augur well for such future applications of DGE ^2^H-MRS in research and in the clinic.

### CRediT authorship contribution statement

**Rui V. Simões:** Conceptualization, Methodology, Investigation, Visualization, Funding acquisition, Project administration, Supervision, Writing – original draft, Writing – review & editing. **Rafael N. Henriques:** Methodology, Writing – review & editing. **Beatriz M. Cardoso:** Investigation, Writing – review & editing. **Francisca F. Fernandes:** Investigation, Writing – review & editing. **Tânia Carvalho:** Methodology, Investigation, Visualization, Writing – review & editing. **Noam Shemesh:** Conceptualization, Methodology, Funding acquisition, Supervision, Writing – review & editing.

## Declaration of Competing Interest

All authors declare that they have no competing interests.
