# Supplementary material for: Glucose fluxes in glycolytic and oxidative pathways detected in vivo by deuterium magnetic resonance spectroscopy reflect proliferation in mouse glioblastoma
Source: Neuroimage Clin. 2022 Jan 5;33:102932. doi: 10.1016/j.nicl.2021.102932 (PMC8760481; doi:10.1016/j.nicl.2021.102932)
Supplement: Supplementary data 1 [file mmc1.docx]

**Supplementary Information**

**Glucose fluxes in glycolytic and oxidative pathways detected *in vivo* by Deuterium Magnetic Resonance Spectroscopy reflect Proliferation in mouse Glioblastoma**

Rui V. Simões^*^, Rafael N. Henriques, Beatriz M. Cardoso, Francisca F. Fernandes,

Tânia Carvalho, Noam Shemesh^*^.

Correspondence to:

[rui.simoes@research.fchampalimaud.org](mailto:rui.simoes@research.fchampalimaud.org);

[noam.shemesh@neuro.fchampalimaud.org](mailto:noam.shemesh@neuro.fchampalimaud.org)

**This document includes:** (page)

Supplementary Methods 2

Supplementary Tables (1 to 4) 5

Supplementary Figures (1 to 6) 10

Supplementary References 17

**Supplementary Methods**

*DGE ^2^H-MRS data averaging*

The SNR constraints associated with the low natural abundance of deuterium impose averaging of DGE ^2^H-MRS data for reliable metabolic quantification. This is demonstrated in **Supplementary Fig. 6-A**, taking tumor G4 as an example. As expected, resampling the data from 8 to 128 averages (NA) led to decreased temporal resolution (from 22.5 sec to 6min, respectively) and increased SNR (from 7 to 22, respectively). Thus, metabolite detection in the original data was more difficult with NA=8, leading to noisy kinetic plots and sub-optimal fits, e.g. for Glx; and while MP-PCA denoising performed similarly in all cases as far as SNR (31-33 range), the metabolic kinetic profiles and their modulation were also compromised when the original data had lower SNR, e.g. for Lac. On the other hand, temporal resolution typically became a limiting factor for the fitting performance when averaging the data beyond NA>64, in both original and denoised data, as evidenced by the respective increase of the confidence intervals. Reproducing this approach for several tumor samples demonstrated empirically that the most reliable fitting performance in both original and denoised data was achieved with NA=64. To evaluate this more objectively, an additional validation step was performed. Specifically, kinetic plots and respective spectra were simulated based on the parameters estimated by model fitting from the real data (taken as ground truth, with NA=64). Then, 3 approaches were tested: (i) adding different noise levels (NL) to the spectra; (ii) changing the total number of spectra in the time-course (equivalent to number of repetitions, NR); or (iii) reproducing the averaging approach used for the real data (in this case from NA= 4 to 256) by adapting NL according to NR to reproduce the SNR profile of the original data. In each case, the data was processed according to the same pipeline (spectral denoising, spectral quantification and kinetic modeling of original and denoised data), and plotted as shown in **Supplementary Fig. 6-B** (sample G4 for consistency). As expected, the performance of the model improved (approaching the ground truth) with NR and decreased with NL. More importantly, when using the averaging approach of the real data, the best performance was achieved within NA= 32 to 64; and slightly better with the latter, hence confirming NA=64 (3min temporal resolution) as the best option for averaging the DGE 2H-MRS data before further processing steps, including MP-PCA denoising.

*Acute hypoxia Protocol*

A total of n=3 C57BL/6j wild-type mice were used to test a protocol for inducing acute hypoxia. The animals were kept under the same conditions of anesthesia (1.5% isoflurane), body heating, and immobilization used for *in vivo* MRI, with the same animal bed, gas vaporizer and oxygen monitoring system (MX300, Teledyne Analytical Instruments, City of Industry CA, USA) to measure the fraction of inspired oxygen (FiO_2_). Mice were shaved in the neck and monitored in real time with *MouseOx*®*Plus* (Starr Life Sciences Corp, Oakmont PA, USA) during regular anesthesia gas (air supplemented with O_2_, to compensate for the lower breathing rate: *FiO_2_* = 31 %) and during acute hypoxia in normobaric conditions (air supplemented with N_2_: *FiO_2_* = 12 %) (Ferrari et al., 2017). Specifically, blood oxygen saturation (*SpO_2_*), breathing rate and heart rate were measured with a mouse collar sensor, and the body temperature with a rectal probe, all monitored in real time with *MouseOx*®*Plus* software for 1 hour in each *FiO_2_* condition, for each animal.

*BOLD T2*-weighted ^1^H-MRI under acute hypoxia*

An additional C57BL/6j wild-type mouse (n=1) was used for *in vivo* monitoring of arterial and brain time-course changes (*ΔR2**) induced by the acute hypoxia protocol, based on BOLD-MRI (Yang et al., 2019), to demonstrate the effectiveness of the acute hypoxia protocol in the brain (**Supplementary Fig. 4**). This was performed on a horizontal 9.4 T BioSpec scanner (Bruker BioSpin, Ettlingen, Germany) equipped with actively shielded gradients (B-GA20S) and an AVANCE III HD console, with 4-element array surface ^1^H cryoprobe (Bruker, Fallanden, Switzerland) actively decoupled from a linear mini-imaging ^1^H resonator with 86 mm inner diameter. After T2-w ^1^H-MRI (*RARE* sequence, x8 acceleration factor, 3000 ms TR, 40 ms TE; 2 averages, 1 mm slice thickness, 70 µm in-plane resolution), BOLD-MRI data were acquired with T2* contrast (multi-gradient echo sequence, 24º flip-angle, 160 ms TR, 1.8 ms TE (20 echos, 5 ms echo-spacing); 1 average, 1 mm slice thickness, 80 repetitions, 156 µm in-plane resolution, 15.5 sec temporal resolution) during *FiO_2_* switch from basal 31% to 12%. T2*-weighted data were processed with MATLAB^®^ R2018b (Natick, Massachusetts: The MathWorks Inc.). T2* maps were calculated pixel-by-pixel based on a logarithmic linear least square fit model, for each time point. The respective *R2** and *ΔR2** maps were then generated (1/T2* and R2*(t)-R2*(baseline), respectively) and ROIs used to plot the average *ΔR2** values at each time point.

*DGE ^2^H-MRS under acute hypoxia*

The Dynamic Glucose-Enhanced ^2^H-MRS protocol (see Material and Methods section 2.3.2 in main article) was repeated with n=2 additional GL261 glioma-bearing mice (GL8-9) under acute hypoxia (FiO_2_=12% in air/ N_2_ mixture).

Supplementary Tables


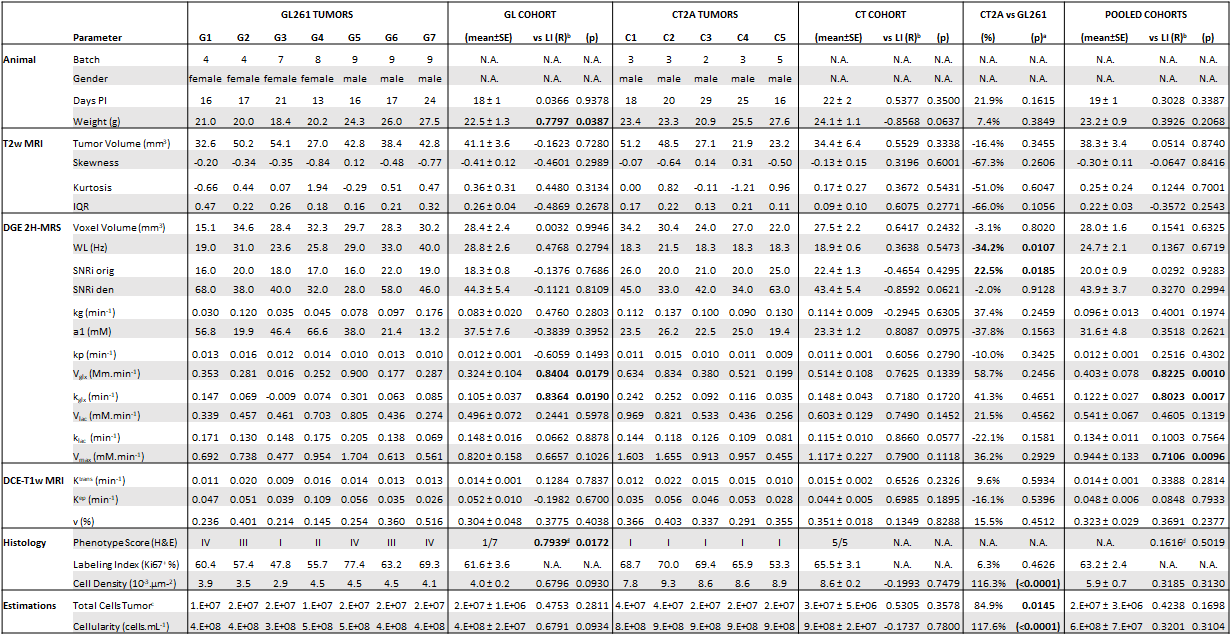


**Supplementary Table 1. Mouse GBM tumor.** Animal information and metrics obtained from multi-modal *in vivo* MRI/MRS and *post-mortem* histopathology and immunostaining.

*K^trans^*, volume transfer constant between plasma and tumor extravascular-extracellular space; *k^ep^*, washout rate between extravascular-extracellular space and plasma; *v*, extravascular-extracellular volume fraction; WL, water linewidth at half-maximum of the water peak; *a1*, Glc concentration after the bolus injection (mM); *kp*, effective rate constant of labeled glucose transfer to tissue (min^−1^); *kg*, apparent rate constant of glucose transfer between blood and tumor (min^−1^); *V_glx_*, maximum rate of Glc consumption for Glx synthesis (mM. min^−1^); *k_glx_*, apparent rate constant of Glx elimination (min^−1^); *V_lac_*, maximum rate of Glc consumption for Lac synthesis (mM. min^−1^); *k_lac_*, apparent rate constant of lactate elimination (min^−1^); *V_max_*, maximum rate of total Glc consumption (mM. min^−1^). ^a^ 2-tailed, unpaired t-Test: highlighted p<0.05; ^b^ Pearson correlation: highlighted rho>0.8 (p<0.05); ^c^ based on the tumor volume (Tv), the average cell density (Cd), and assuming an average GL261 cell radius of 10 µm (Cr, (Roberts et al., 2020)): Tv x Cd x 10^9^/ Cr; ^d^ Prevalence of score I (n/Total); ^e^ Kendall’s rank correlation (Tau).

**
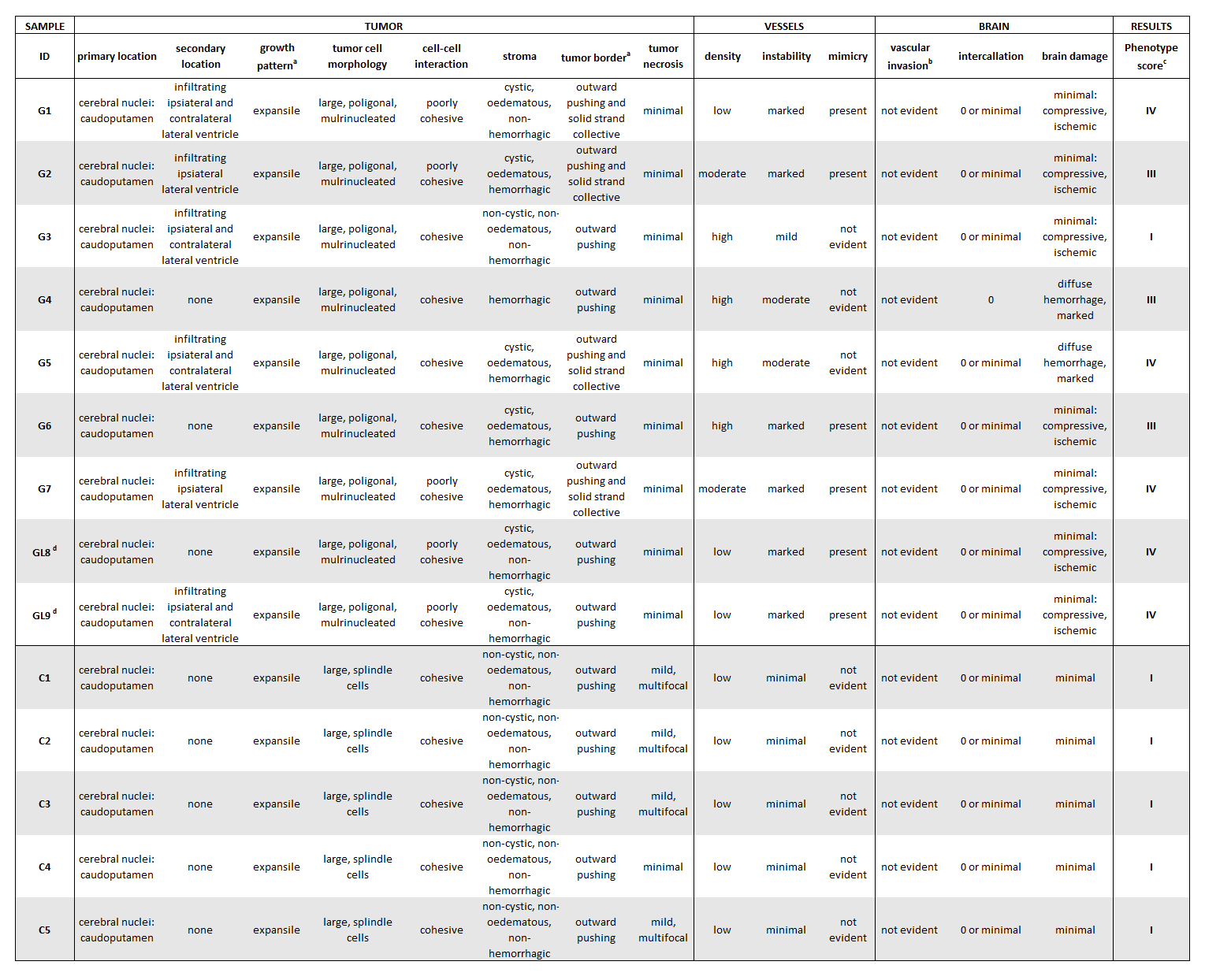
**

^a^ predominant; ^b^ 0 (absent) or 1 (present); ^c^ progression phase; d tumors studied *in vivo* under acute hypoxia.

**Supplementary Table 2. Histopathologic evaluation.** Analysis of H&E histologic sections from each tumor and scoring according to phenotypic features of their stromal-vascular fraction.

**Supplementary Table 3. DGE ^2^H-MRS of GL261 tumors under acute hypoxia.**


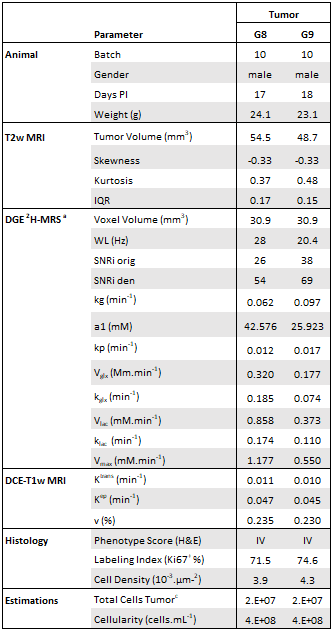


^a^ *FiO2* = 12%

**Supplementary Table 4. Differences in voxel vs tumor volumes do not correlate significantly to glucose oxidation or glycolytic rates within each cohort or across pooled cohorts.**


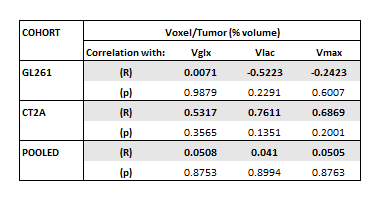


Supplementary Figures


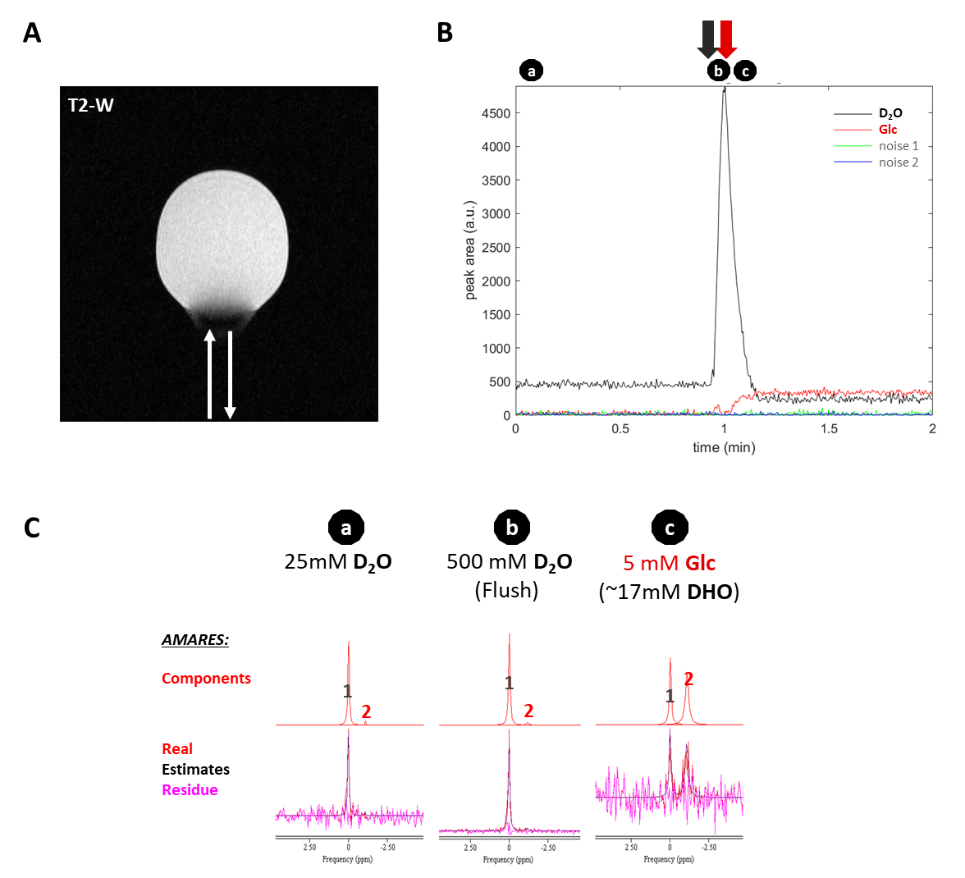


**Supplementary Figure 1. Evaluation of spectral quality and sensitivity of dynamic ^2^H-MRS *in vitro*.** **A** T2-w RARE image of a homebuilt glass-sphere phantom initially filled with 25 mM D_2_O solution and connected through an injection line (filled with 500 mM D_2_O, dead volume) to a syringe loaded with 5 mM 6,6′-^2^H_2_-glucose in saline (Glc, with DHO natural abundance ~17 mM). **B** Time-course acquisition of ^2^H-MRS data (*pulse-acquire* sequence, 67 º flip angle, 1 average, TR=300ms, 200 repetitions, 512 spectral points, and 1749 Hz spectral width) in different conditions: (a) 25 mM D_2_O; (b) during flush with 500 mM D_2_O (black arrow); and (c) after injection of 5 mM ^2^H-Glc (red arrow, following the flush in (b)). **C** Spectral quality (real) obtained in the 3 conditions (a, b, c) and AMARES quantification of deuterated water and glucose peaks (components 1 and 2, respectively; residuals of the fitting in fuchsia). The results show good sensitivity to DHO natural abundance (~0.03% in water) and physiologic concentrations of glucose (Glc 5 mM), and overall time-course spectral stability.


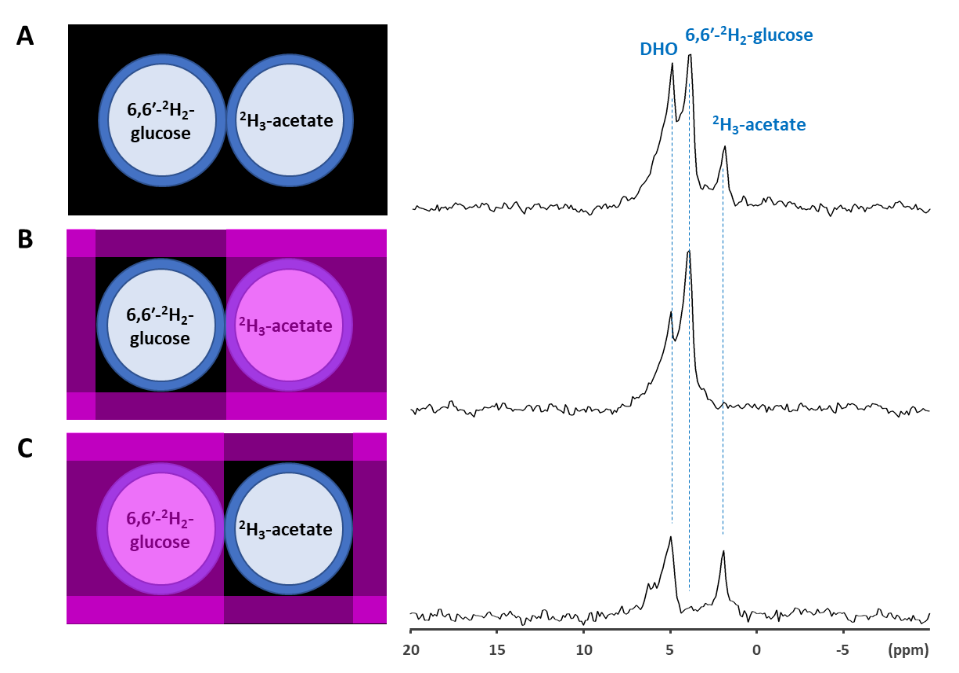


**Supplementary Figure 2. Evaluation of ^2^H-MRS with outer volume suppression *in vitro*.** A 2-compartment phantom was filled with: 20 mM 6,6′-^2^H_2_-glucose in PBS; and 20 mM ^2^H_3_-acetate in PBS. ^2^H-MRS acquired with a *pulse-acquire* sequence, 175ms TR, 256 points, 1749 Hz, 55º flip angle. **A** No outer volume suppression (OVS). **B-C** OVS applied outside the phantom, with 6 pulses (10mm slabs), fully covering the acetate compartment (**C**), or the glucose compartment (**C**), demonstrating the good performance of volume selection for ^2^H-MRS.


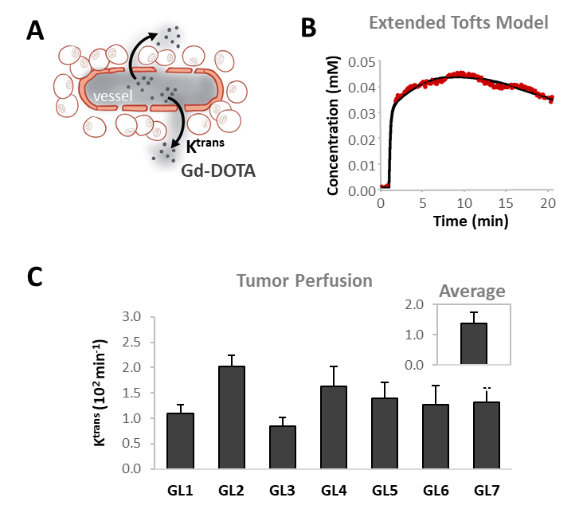


**Supplementary Figure 3. Assessment of GL261 tumor perfusion based on DGE T1-MRI *in vivo*. A** Model for transfer Gd-DOTA from the vascular compartment to the intratumor-extracellular space (K^trans^, min^-1^). **B** Extended-Tofts for kinetic modelling (GL2 displayed). **C** Perfusion estimates for all the tumors.

**
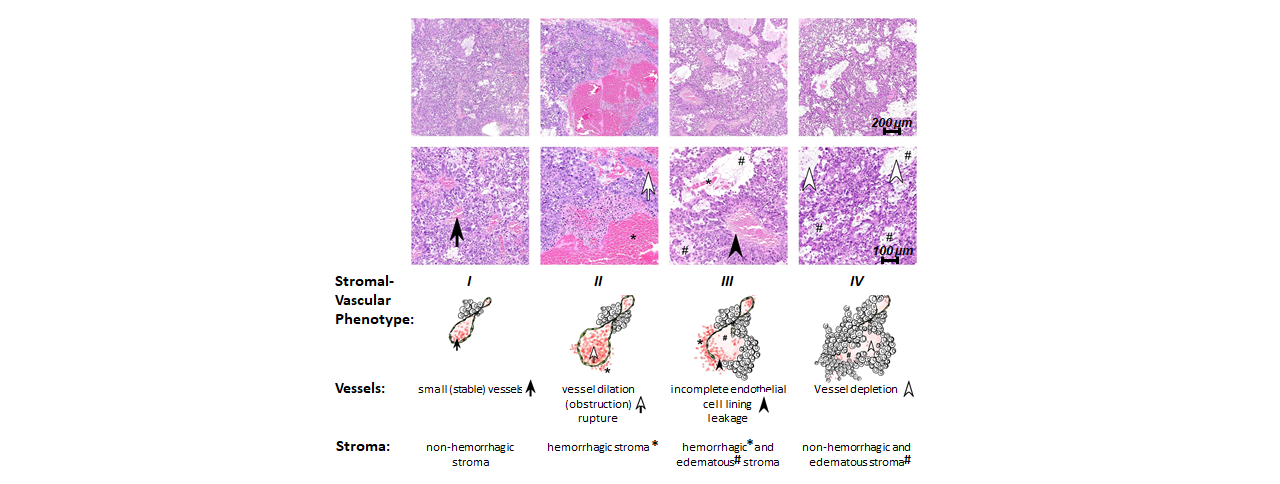
**

**Supplementary Figure 4. Individually tumor scoring of stromal-vascular phenotypes.** Distinct morphological features of the stromal-vascular fraction in different GL261 tumors, showing four phenotypes: I, small vessels, complete endothelial cell lining and sparse hemorrhages; II, vasodilation and marked multifocal hemorrhages; III, necrosis of the vascular wall, incomplete endothelial cell lining, vascular leakage, and edematous stroma; and IV, vascular depletion and edematous stroma.


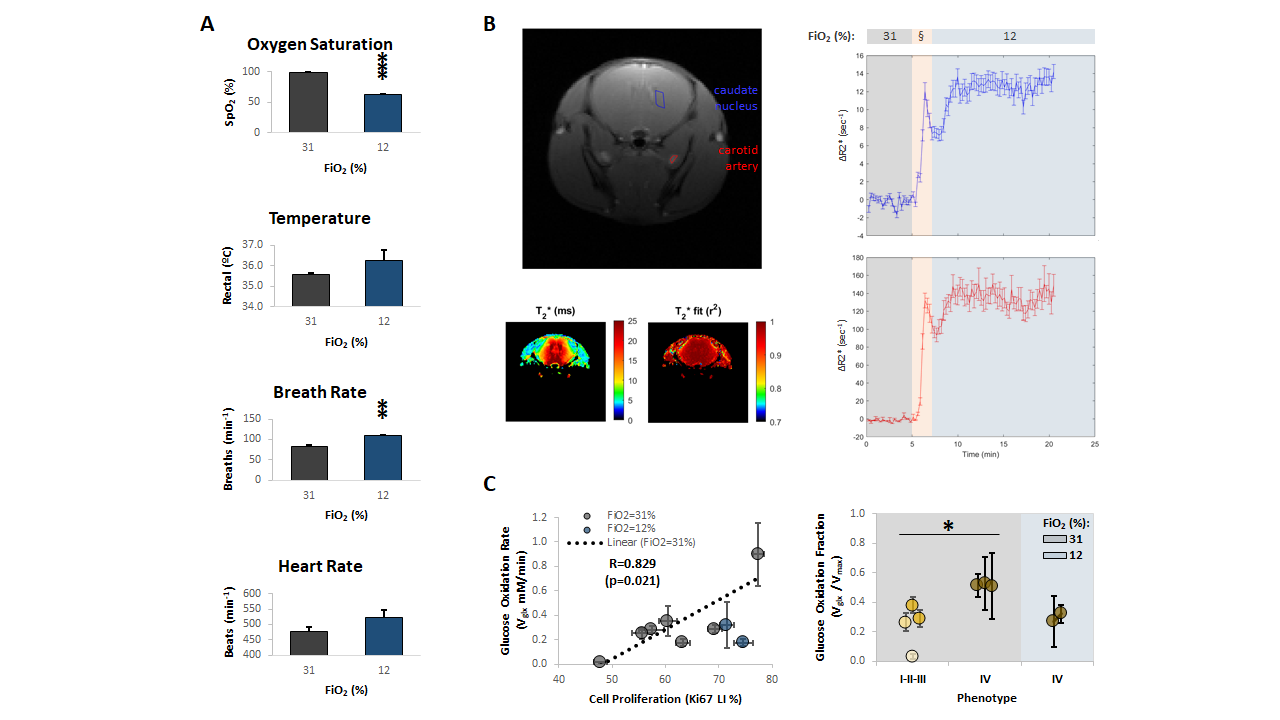


**Supplementary Figure 5. Effect of acute hypoxia on glucose metabolism of GL261 *in vivo*. A** Physiologic bench monitoring of n=3 WT mice with the *MouseOxPlus*® mouse collar sensor, kept under the same conditions of immobilization, anesthesia, and body heating used for MRI (same animal holder). Two conditions tested, with fraction of inspired oxygen (*FiO_2_*): 31% and 12%, under normobaric conditions. Time-course measurements of oxygen saturation (*SpO*_2_), rectal temperature, breath and heart rate are displayed, plotted as average values from different animals (left-side). **B** Dynamic monitoring of relative brain oxygenation with BOLD T2* MRI, under the two *FiO_2_* conditions in n=1 WT mouse: left-side-top, reference image (first echo under basal condition, 31% FiO_2_) with ROIs overlaid (red, carotid artery; blue, caudate nucleus); left-side-bottom, respective T2* map (left-side) and r^2^ goodness-of-fit map (right-side); *ΔR2** time-course plots for each ROI (caudate nucleus (top, blue), and carotid artery (bottom, red)) under different FiO_2_ conditions (1-5 min (grey, basal condition), 31%; 5-7 min (orange, gas adjustment period), from 31 to 12% (§); 7-20min (dark blue, acute hypoxia period), 12%). The results show increased *R2** relaxivity under lower oxygenation, consistent with a higher deoxyhemoglobin arterial fraction (red ROI) leading to acute hypoxia in the brain parenchyma (blue ROI). **C** DGE ^2^H MRS in n=2 GL261 glioma-bearing mice under hypoxia (G8-9, 12% FiO_2_): left-side, deviation from the *V_glx_* vs. *Ki67-LI* linearity in the main GL261 cohort under regular anesthesia conditions (G1-7, 31% FiO_2_: R=0.829, p=0.021); right-side, significantly higher glucose oxidation fraction (*V_glx_/V_max_,* estimate±SE) in GL261 tumors with phenotype IV (n=3) compared to I-II-III (n=4; p=0.026) during regular anesthesia (31% FiO_2_) not observed in phase IV tumors during acute hypoxia (12% FiO_2_). Plots: mean±SE. ** p<0.01, *** p<0.001.


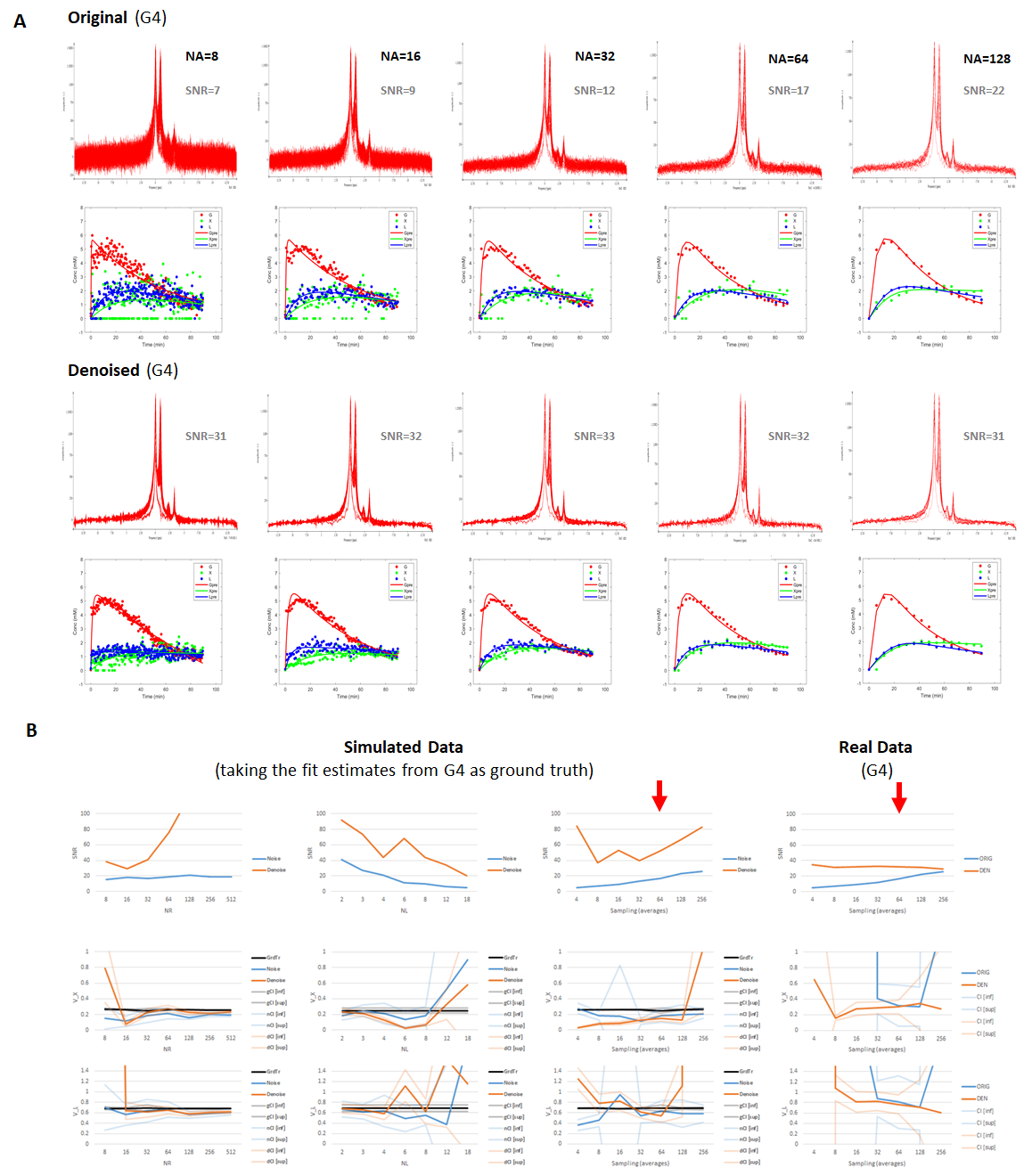


**Supplementary Figure 6. Effect of averaging DGE ^2^H-MRS data for MP-PCA denoising.** Tumor G4 data displayed as an example. **A** Resampling the data from 8 to 128 averages (NA) did not impact MP-PCA denoising performance with regards to SNR improvement, but impacted metabolic kinetic profiling and modulation (e.g. Lac at NA=8 vs. 64). **B** Simulated and real data (ground truth). Three approaches tested with simulated spectra: (first-from-left) adding different noise levels (NL) to the spectra; (second-from-left) changing the total number of spectra in the time-course (equivalent to number of repetitions, NR); or (third-from-left) reproducing the averaging approach used for the real data (forth-from-left) by adapting NL according to NR to reproduce the SNR profile of the original data, in this case from NA= 4 to 256. Best performance highlighted (NA= 64, red arrow).

**Supplementary References**

Ferrari, M., Jain, I.H., Goldberger, O., Rezoagli, E., Thoonen, R., Cheng, K.H., Sosnovik, D.E., Scherrer-Crosbie, M., Mootha, V.K., Zapol, W.M., 2017. Hypoxia treatment reverses neurodegenerative disease in a mouse model of Leigh syndrome. Proc Natl Acad Sci U S A 114, E4241-E4250. DOI:10.1073/pnas.1621511114.

Roberts, T.A., Hyare, H., Agliardi, G., Hipwell, B., d'Esposito, A., Ianus, A., Breen-Norris, J.O., Ramasawmy, R., Taylor, V., Atkinson, D., Punwani, S., Lythgoe, M.F., Siow, B., Brandner, S., Rees, J., Panagiotaki, E., Alexander, D.C., Walker-Samuel, S., 2020. Noninvasive diffusion magnetic resonance imaging of brain tumour cell size for the early detection of therapeutic response. Sci Rep 10, 9223. DOI:10.1038/s41598-020-65956-4.

Yang, D.M., Arai, T.J., Campbell, J.W., 3rd, Gerberich, J.L., Zhou, H., Mason, R.P., 2019. Oxygen-sensitive MRI assessment of tumor response to hypoxic gas breathing challenge. NMR Biomed 32, e4101. DOI:10.1002/nbm.4101.
